# Protease Activity of PprI Facilitates DNA Damage Response: Mn(2+)-Dependence and Substrate Sequence-Specificity of the Proteolytic Reaction

**DOI:** 10.1371/journal.pone.0122071

**Published:** 2015-03-26

**Authors:** Yunguang Wang, Qiang Xu, Huiming Lu, Lin Lin, Liangyan Wang, Hong Xu, Xianyan Cui, Hui Zhang, Tingting Li, Yuejin Hua

**Affiliations:** 1 Key Laboratory of Chinese Ministry of Agriculture for Nuclear-Agricultural Sciences, Institute of Nuclear-Agricultural Sciences, Zhejiang University, Hangzhou, China; 2 Zhejiang Key Laboratory of Radiation Oncology, Zhejiang Cancer Research Institute, Zhejiang Cancer Hospital, Hangzhou, China; 3 National Institute on Aging, Biomedical Research Center, National Institutes of Health, Baltimore, United States of America; The University of Hong Kong, HONG KONG

## Abstract

The extremophilic bacterium *Deinococcus radiodurans* exhibits an extraordinary resistance to ionizing radiation. Previous studies established that a protein named PprI, which exists only in the *Deinococcus-Thermus* family, acts as a general switch to orchestrate the expression of a number of DNA damage response (DDR) proteins involved in cellular radio-resistance. Here we show that the regulatory mechanism of PprI depends on its Mn(2+)-dependent protease activity toward DdrO, a transcription factor that suppresses DDR genes’ expression. Recognition sequence-specificity around the PprI cleavage site is essential for DNA damage repair *in vivo*. PprI and DdrO mediate a novel DNA damage response pathway differing from the classic LexA-mediated SOS response system found in radiation-sensitive bacterium *Escherichia coli*. This PprI-mediated pathway in *D*. *radiodurans* is indispensable for its extreme radio-resistance and therefore its elucidation significantly advances our understanding of the DNA damage repair mechanism in this amazing organism.

## Introduction

DNA repair is an essential process for cells to maintain their genomic stability. Cells have evolved elaborate systems to recover its DNA from damage caused by hazardous environmental stresses. The Gram-positive bacterium *Deinococcus radiodurans* is one of the commonly used model organism for studying DNA repair due to its exceptional tolerance to extensive DNA damage inflicted by various DNA-damaging agents such as ionizing radiation (IR), ultraviolet light (UV), chemical mutagens and desiccation [1–4].

PprI (also called IrrE), a protein that is unique to the *Deinococcus-Thermus* family, has been identified as one of the essential proteins for the DNA damage response and repair processes [5, 6]. Disruption of the *pprI* gene renders the bacteria sensitive to various DNA damage. It has been observed that PprI can bind to the promoter regions of *recA* and *pprA*, two genes important for DNA repair [7–10]. Comparison between the wild type strain and *pprI*-knockout mutant confirms that PprI regulates the expression of a series of genes, including those involved in the DNA repair process [7, 11]. Crystal structure suggests that PprI possesses three structure domains: a zinc peptidase-like domain, a helix-turn-helix motif and a GAF-like domain [12]. Mutants designed to disrupt this putative protease activity sensitize the bacteria towards UV and ionizing radiation [12], indicating that the protease activity might be important for the function of PprI. However, the mechanism by which PprI regulates gene expression was not clear and the substrate of its protease activity has not been identified until recently.

DdrO, a putative XRE (xenobiotic response element) family transcription regulator, has been predicted to play a role in DNA damage response as it is one of the few transcription regulators which are up-regulated following radiation damage [13, 14]. Recently, Ludanyi *et al* [15] reported that PprI can cleave DdrO in the closely related *Deinococcus deserti*. They first detected the interaction between PprI and DdrO using a bacterial two hybrid method and far western method. Then they observed that PprI can cleave DdrO *in vitro* and when co-expressed in *Escherichia coli*. The cleavage of DdrO in *D*. *deserti* was found to occur after irradiation. While unable to detect the binding of DdrO to the promoter region of DNA damage response (DDR) genes directly, Ludanyi *et al* used a reporter gene approach to show that DdrO can regulate these genes.

Unaware of the results in *D*. *deserti* at the time, we came to essentially the same conclusion independently regarding PprI’s cleavage of DdrO in *D*. *radiodurans*. In addition to what have been reported by Ludanyi *et al*, we discovered that this protease activity is Mn(2+) dependent. Furthermore, in this report, the direct binding of DdrO to DDR gene promoter regions were confirmed with gel mobility shift assay. The sequence specificity of the proteolytic reaction was further explored and mutations that can render DdrO uncleavable were identified. Replacing the native DdrO with the uncleavable mutant dramatically sensitized *D*. *radiodurans* toward various DNA damage. These results further clarify the mechanism by which DNA damage response were regulated in this amazing organism.

## Materials and Methods

### Materials

Unless otherwise stated, standard chemicals were of high purity available from Sigma-Aldrich. Tryptone, yeast extract were purchased from Oxford. Enzymes for molecular biology were from Takara. HiTrap Heparin HP column and Superdex 75 were from GE healthcare.

### Strains and culture

All plasmids and strains were listed in S1 Table. *D*. *radiodurans* strains were grown at 30°C in TGY broth (0.5% tryptone, 0.3% yeast extract, 0.1% glucose) or on TGY plates with 1.5%(w/v) agar powder. *E*. *coli* strains, containing DH5α and BL21(DE3), were cultivated in LB broth (1% tryptone, 0.5% yeast extract, 1% NaCl) or on LB medium with 1.5%(w/v) agar at 37°C. Antibiotics used to select *E*. *coli* were kanamycin (50 μg/ml) and ampicillin (50 μg/ml). For *D*. *radiodurans*, concentrations of kanamycin, chloramphenicol and streptomycin were 10 μg/ml, 4 μg/ml, 10 μg/ml, respectively.

### Protein expression and purification

All the interested gene fragments obtained by PCR amplification (primers listed in S2 Table) were cloned into pET28-HMT vector, which is a modified pET28a vector with a N-terminal hexahistidine tag followed by an additional maltose binding protein (MBP) tag and a tobacco etch virus (TEV) protease site before the gene of interest. The resulting plasmids were transformed into *E*. *coli* BL21(DE3) for overexpression of 6xHis-MBP tagged proteins, respectively. The expression strains were grown in LB broth containing 30 μg/ml kanamycin at 37°C to exponential phase. 0.5 mM isopropyl-β-D-thiogalactoside (IPTG) was added when OD_600_ reached 0.6. Induced expression lasted for 4 hours before the cells were harvested by centrifugation. For purification, cells were washed twice with phosphate saline and re-suspended in lysis buffer (500 mM NaCl, 20 mM Tris-HCl pH 8.0, 1000 units/ml DNaseI, 1 mM PMSF, 1 mM EDTA), then disrupted using a High Pressure Cell Cracker (JNBio). Cell debris was removed by centrifugation (20,000g for 30 min at 4°C). An AKTA Purifier system was used for purification. The supernatant was loaded onto a Ni-NTA superflow column. The unbound impurity was removed using 3 column volumes of buffer A (500 mM NaCl, 20 mM Tris–HCl pH 8.0, 30 mM imidazole). The protein was then eluted with buffer B (500 mM NaCl, 20 mM Tris-HCl pH 8.0, 300 mM imidazole). The collected sample was flown through an amylose column (NEB), eluted by 500 mM NaCl, 20 mM Tris–HCl pH 8.0 and 10 mM maltose. Cleavage of the 6x His-MBP tag with a His-tagged TEV protease was carried out at 15°C overnight. After short centrifugation, the supernatant was purified by Ni-NTA column and MBP column again to discard the 6xHis-MBP tag. NaCl concentration of the sample was lowered to 100 mM by dialysis. Further purification was carried out using a HiTrap Heparin HP column (GE healthcare). A gradient elution from 100 mM to 600 mM NaCl was applied. Final samples were analyzed using gel filtration chromatography (Superdex 75) and SDS-PAGE.

### Site–directed mutagenesis *in vitro*

Site directed mutagenesis was carried out using the pET28-HMT expression plasmid containing *pprI* or *ddrO* gene as template (primers listed in S2 Table). Amplification was performed using PrimeSTARHS DNA polymerase (Takara). Following temperature cycling, the amplified product was treated with DpnI, then transformed into DH5α. The clones were screened on LB plates with kanamycin. After validation by DNA-sequencing, the mutant vectors were further transformed into BL21(DE3) for expression.

### Construction of site-mutant strain of *D. radiodurans*

The mutant of *ddrO*-R109E *in vivo* was constructed as previously described [16] with modification (S1A Fig.). In short, *ddrO*-R109E gene in pET28-HMT vector and downstream fragment of *dr2574* in strain R1 were amplified by appropriate primers (S2 Table) and then digested with BamHI and HindIII, respectively. The reaction products were purified by Gel Extraction Kit (Qiagen) and then were ligated with streptomycin-resistance gene containing BamHI and HindIII cohesive ends. The ligation product was used as the template for PCR. The amplified product was transformed into exponential-phase wild type R1 cells with CaCl_2_ treatment. Mutant (named MR109E) was selected on TGY plates with streptomycin (10 μg/ml) and validated by PCR and sequencing (S1B and S1C Fig.).

### Complementation of *ddrO* or *pprI*

The complementary plasmids and strains were shown in S1 Table. Shuttle plasmid pRADK was used to construct complementary plasmid. The resulted plasmids containing pRADK-*ddrO*, pRADK-*pprI* and pRADK-*pprI*(H118L) were transformed into the mutant strains MR109E and YR1, respectively. Clones were screened on TGY plates with different antibiotics.

### Time course experiments after gamma radiation

Wild type R1, YR1, MR109E and CMR109E strains were cultivated in TGY culture to late exponential phase (OD_600_ = 1.0). A fraction of each culture was saved as control at room temperature while the rest was exposed to 2 kGy radiation at the same temperature. Then all samples were incubated at 30°C. Cells were harvested from equal volume of culture at different time points, and re-suspend and washed twice by PBS prior to lysis by sonication on ice [7].

### Western blotting

Western blot assay was performed as previously described [17]. The proteins were separated using gel electrophoresis, then transferred to PVDF membranes (Millipore). Membranes were incubated in PBS with 0.1% Tween-20 (PBST), 5% skim milk powder for 60 minutes at room temperature, then washed five times using PBST. Rabbit anti-DdrO polyclonal antibody, obtained using purified DdrO as antigen, was applied to detect the DdrO protein on the membranes. After washing off the nonspecific bound DdrO antibody on the PVDF, Goat anti-rabbit IgG conjugate HRP (Sigma) was added as the secondary antibody and the antibody binding was detected using a colorimetric reaction [5].

### RNA extraction and real-time quantitative PCR (QRT-PCR)

Wild type R1 and mutants were cultivated in TGY to mid-exponential phase (OD_600_ = 0.5). For time course of gamma radiation treatment, sample preparation was carried out as described above. RNA extraction and QRT-PCR were performed as previously described [16]. Briefly, Total RNA was purified from cells using a RNA Purification Kit (Qiagen) followed by RNaseA free DNaseI (NEB) treatment. Nano-drop (Thermo) was used to detect RNA quality and concentration. For QRT-PCR, *dr0089*, a house-keeping gene, was used as an internal control, because its expression *in vivo* was not influenced by normal treatments. Seven genes were quantified by QRT-PCR (primers listed in S2 Table) on a Stratagene MX3005.

### Metal ion removal

The protein samples were dialyzed using dialysis membrane (Amersco, 8000-14000Da) in 200 mM NaCl, 20 mM Tris-HCl pH 8.0, 0.5 mM EDTA at 4°C for 2 hours. Then the EDTA in protein samples was removed by dialysis in 200 mM NaCl, 20 mM Tris-HCl pH 8.0 for 2 hours.

### PprI cleavage activity detection *in vitro*

The protease activity of PprI was performed *in vitro* by incubating 450 μM DdrO with 8 µM PprI at 37°C for 30 minutes unless otherwise specified. The final reaction buffer was 200 mM NaCl, 20 mM Tris-HCl pH 8.0, 15% glycerol, 1 mM DTT, 1 mM MnCl_2_ or other metal ion at the same concentration. SDS–PAGE was applied to detect the cleavage result.

### Chromatin-immunoprecipitation (ChIP)

ChIP assays were performed as described [18, 19] with appropriate modifications. Briefly, *D*. *radiodurans* cells were collected in TGY culture at late exponential phase, and then washed twice by PBS. 1% of formaldehyde was added to crosslink DNA-protein *in vivo*. After 25 minutes, reactions were quenched by the addition of 0.125 M glycine. Cells were then harvested by centrifugation, washed twice with Tris-buffered-saline (pH 7.5), and re-suspended in lysis buffer (160 mM NaCl, 20 mM Tris-HCl pH 7.5, 1 mM EDTA, 1x Roche Protease Inhibitor Cocktail). Cells were disrupted, and DNA was sheared by a High Pressure Cell Cracker to an average size of 200 to 700-bp at 4°C. After centrifugation, the supernatant was moved to sterile microcentrifuge tubes in 2ml aliquots. The sample was then incubated with 50μl of EZ-CHIP^TM^ protein G beads (Millipore) and 10mg of normal rabbit IgG as the negative control or DdrO rabbit polyclonal antibody for 4 hours at 4°C with agitation. Magnetic field was applied and the beads were washed by re-suspension in 1 ml each of the cold washing buffers (Millipore). 500 μl of elution buffer with RNaseA was added to each tube containing beads followed by incubation at 65°C for 15 minutes. The eluted samples was collected after centrifugation and incubated at 65°C overnight to reverse the cross-linking of protein-DNA complexes in order to free the DNA. Proteinase K was added to degrade the protein. Then DNA was purified by Gel Extraction Kit (Qiagen). QRT-PCR was applied to analyze the relative abundance of seven chosen DDR genes using immunoprecipitated DNA.

### Survival measurement after gamma, UV radiation and H_2_O_2_ treatment

The methods for the survival measurements have been reported previously [7]. Briefly, for gamma radiation sensitivity assay, *D*. *radiodurans* cells in late exponential phase (OD_600_ = 1.0) were suspended in 10 mM MgSO_4_ and irradiated by ^137^C_S_-γ ray (Dose rate: 1 kGy/h) at several different dosages (0, 2, 4, 6 kGy). For UV radiation, cells were diluted, plated on TGY plates, and exposed to UV-254nm at different dosages (0, 48, 84, 120, 168 J/m^2^). For H_2_O_2_ effect, the diluted cells were treated by H_2_O_2_ at different concentration (0, 10, 20, 30 mM). Cells plated on the TGY plates were incubated at 30^°^C for 3 days before colony counting.

### Gel mobility shift assay (GMSA)

Promoter regions of DDR genes were amplified by PCR (primers listed in S2 Table) for GMSA. The final reaction mixture is composed of 150 mM NaCl, 20 mM Tris-HCl pH 8.0, 5 mM MgCl_2_, 1.6 μM DNA and protein at 0, 6, 8, 12, 16 μM. The mixture was incubated at 30^°^C before loading onto 12% TB-PAGE and run for 2 hours. The bands were visualized on a Typhoon scanner (GE healthcare).

### Peptide mapping and C-terminal sequencing

The peptide mapping work was carried out by using the NanoLC-MS/MS peptide sequencing technology. In brief, each protein sample was digested with sequencing grade modified trypsin and chymotrypsin. The resulted peptides mixture was analyzed by a LC-MS/MS system (ABI), in which a high performance liquid chromatography (ABI) with a 75 micrometer inner diameter reverse phase C18 column was on-line coupled with an ion trap mass spectrometer. The mass spectrometric data acquired were used to search the most recent non-redundant protein database with ProtTech’s proprietary software suite.

## Results

### Mn(2+)-dependence of PprI-mediated cleavage

**Fig 1:**
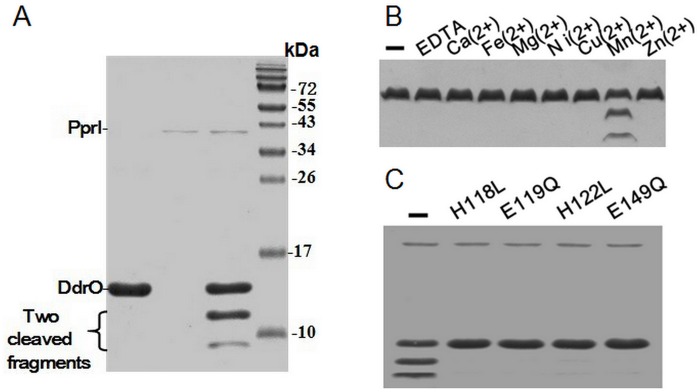
Metal dependence of PprI protease. (A) PprI cleavage activity *in vitro* was measured using purified PprI and DdrO protein. PprI cleaves DdrO into two fragments as indicated by the SDS-PAGE gel. (B) Metal ion scan for the protease activity. From left to right: without additive, 1 mM EDTA, 1 mM CaCl_2_, 1 mM FeCl_2_, 1 mM MgCl_2_, 1 mM NiSO_4_, 1 mM CuCl_2_, 1 mM MnCl_2_, 1 mM ZnCl_2_. Mn(2+) is necessary for the protease activity. (C) Mutations at the metal-binding sites of PprI abolish the proteolytic activity. From left to right: wild type PprI, H118L mutant, E119Q mutant, H122L mutant and E149Q mutant. These mutations were chosen based on the crystal structure from *D*. *deserti* (PDB code 3DTI) [9].

Running the reaction mixture of PprI and DdrO on SDS-PAGE gel shows that two lower molecular weight bands emerge while the DdrO band becomes weaker (Fig. 1A). Mass spectra analysis identified both of the two newly produced bands as fragments of DdrO with a molecular weight of 12214.1 Da and 3002.6 Da, respectively (S2 Fig.), demonstrating that the fragments are the products of cleavage at a specific site. This result is identical to what has been reported in *D*. *deserti* [15]. The N-terminal domain of the *D*. *deserti* PprI structure adopts a zinc metallopeptidase fold [12]. A zinc ion was found in the structure to be coordinated by His-82, Asp-83, His-86 and Asp-113. To test the metal requirement of the cleavage activity for *D*. *radiodurans* PprI, residual divalent metal cation was first removed from the purified PprI by EDTA. After a second dialysis to get rid of EDTA, various metal ions were added to the PprI solution 1 hours before the reaction was initiated by the addition of purified DdrO. As shown in Fig. 1B, the EDTA treatment abolishes protease activity of PprI and among the seven types of common divalent metal ion tested, Mn(2+) is the only one that restores this proteolytic capability. No detectable protease activity is observed for other metal ions even with higher metal concentration (as high as 5 mM) or longer incubation and reaction time (S3A Fig.). 1mM MnCl_2_ alone does not cause any visible degradation of DdrO (S3B Fig.). This is interesting as *D*. *radiodurans* was found to accumulate high concentration of Mn(2+) in the cell [16], which was suggested to be important for protection of protein against free radical created by radiation. In addition, mutation of residues mediating the metal binding (His-118, Asp-119, His-122, Asp-149) abolished or reduced the cleavage activity, which further confirmed the necessity of metal ion for the PprI function (Fig. 1C). These data indicate that the proteolytic reaction of PprI is dependent on Mn(2+).

### Recognition sequence specificity of PprI cleavage

**Fig 2:**
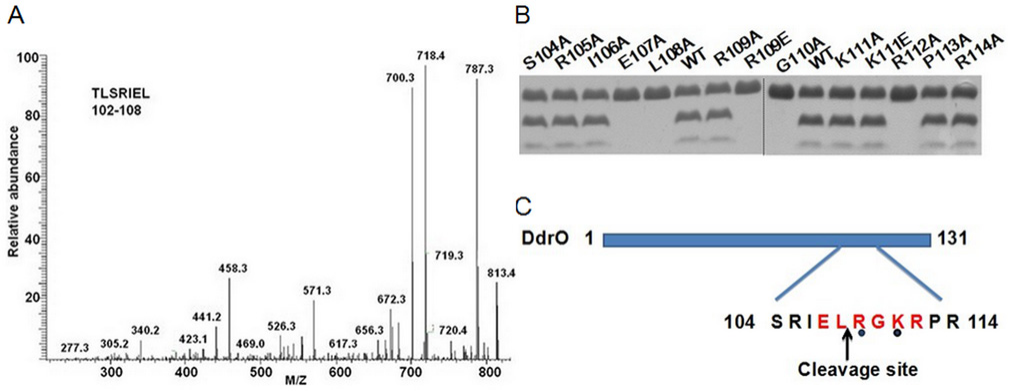
Recognition sequence specificity of PprI protease. (A) C-terminal sequencing of the larger cleaved fragment. The peptide mapping work was carried out using the NanoLC-MS/MS peptide sequencing technology. C-terminal peptide was identified: TLSRIEL (102–108) based on tryptic digestion. (B) Alanine scan at the DdrO cleavage site was conducted to assay the sequence specificity. Arg-109 and Lys-111 were also mutated to Glu. (C) The sequence specificity requirement is summarized here. Amino acid residues in red are essential to recognition and cleavage of the protease. The residues marked by black dots are variable.

According to the molecular mass detecting (S2 Fig.) and C-terminal sequencing of enzymatic product (Fig. 2A), the proteolytic cleavage site is between Leu-108 and Arg-109. This is same as what has been reported for *D*. *deserti*. To test the sequence specificity of PprI cleavage, a series of DdrO mutants were constructed. Mutation of residues Glu-107, Leu-108, Gly-110 or Arg-112 to Ala separately makes these mutants uncleavable to PprI, while the R109A and K111A mutants can still be cut (Fig. 2B). To test whether more drastic changes can be tolerated at the cleavage site, Arg-109 and Lys-111 were mutated to the acidic Glu residue. Although PprI could still cleave the K111E mutant, it failed to cut the R109E mutant. These results were summarized in Fig. 2C. The amino acid sequence near the cleavage site is strictly conserved in all DdrO homologs identified (S4 Fig.). These results suggest that the proteolytic activity of PprI is likely to be sequence-specific.

### The protease substrate DdrO binding to DDR promoter regions

**Fig 3:**
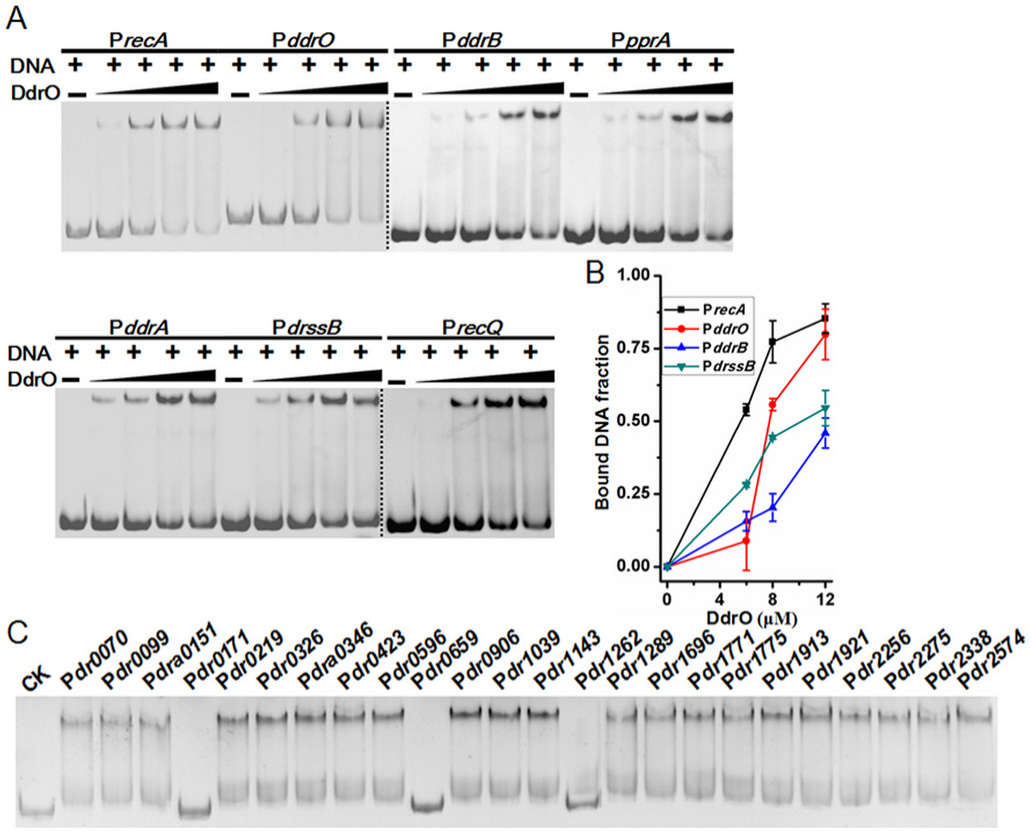
DdrO binds to the promoter regions of DDR genes *in vitro*. (A) Gel mobility shift assay using purified, tag removed DdrO and DDR gene promoter regions. The concentration of DNA substrates is 1.6 μM and the DdrO concentrations are 0, 6, 8, 12, and 16 μM, respectively. (B) Analysis of the interaction between DdrO and promoter regions. DNA band density on the GMSA gel was analyzed by Quantity One software. And the data represent the average of three independent experiments. (C) Detecting the DdrO binding capability of RDRM containing promoter regions. CK was a blank control without adding DdrO.

**Fig 4:**
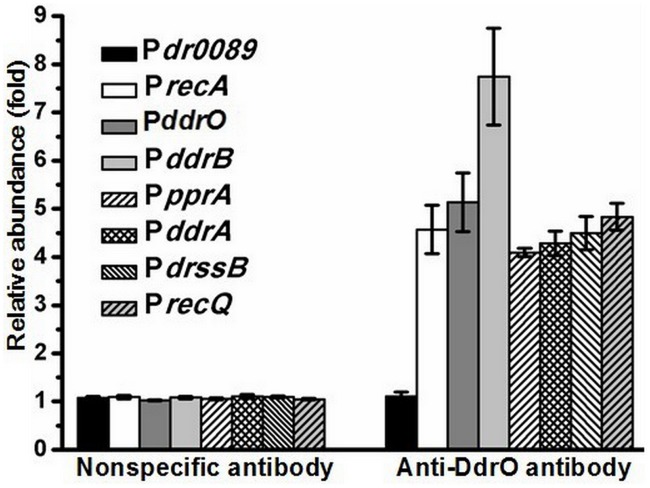
The interaction between DdrO and the promoter regions *in vivo*. QRT-PCR was performed using the immunoprecipitated DNA. DNA fragments cross-linked to DdrO were enriched by rabbit anti-DdrO antibody. Nonspecific normal antibody of rabbit in ChIP assay was applied as a blank control. *Dr0089* was used as an internal blank in QRT-PCR.

**Fig 5:**
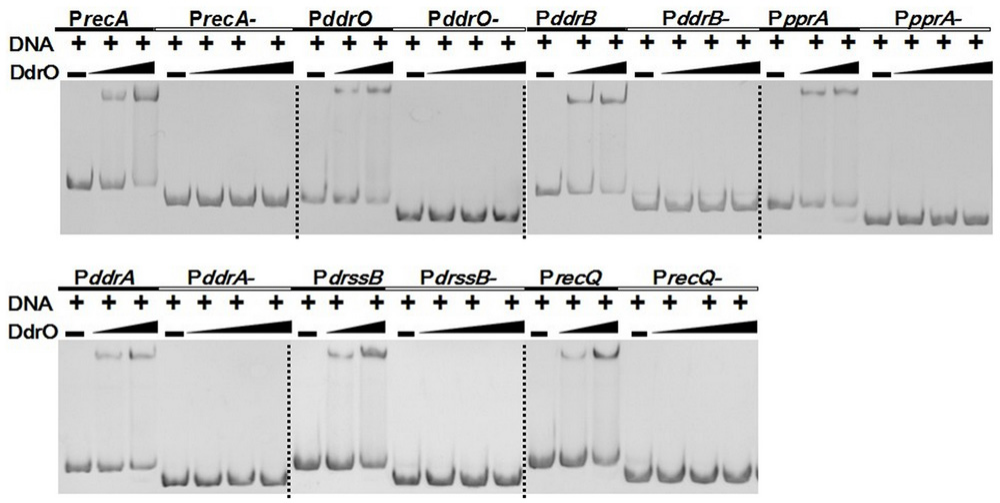
RDRM sites in the promoter regions were imperative for the binding of DdrO. The promoter regions (P*recA-*, P*ddrO-*, P*ddrB-*, P*pprA-*, P*ddrA-*, P*drssB-*, P*recQ-*) absent the RDRM sequence fail to bind DdrO.

**Fig 6:**
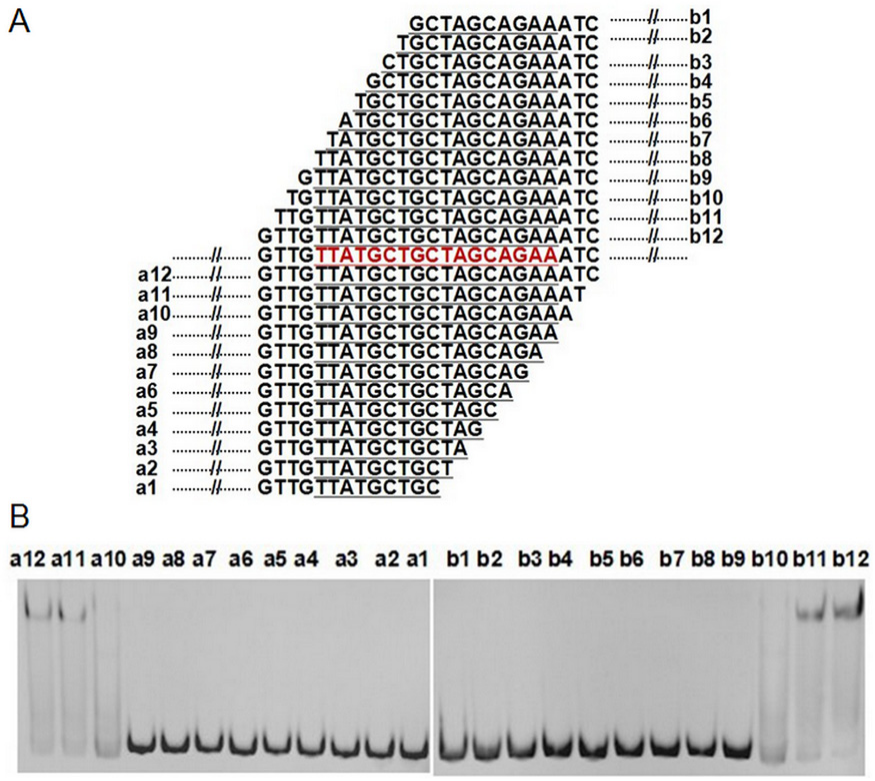
Identification of the minimum DdrO binding sequence. (A) A series of DNA double strand candidates for detection. All the sequences around the RDRM site of P*recA* were obtained by PCR amplification. RDRM site is colored in red and underlined. (B) Identification of the interaction between DdrO and the above sequences through GMSA.

Using gel mobility shift assay (GMSA), we observed that purified DdrO has the capability to bind to the promoter regions of *recA* (*dr2340*) and *pprA* (*dra0346*), which are also the binding targets of PprI [7]. Further study showed that DdrO can also bind to the promoter regions of the reported DNA damage response genes (DDR) *in vitro*, including *ddrB* (*dr0070*), *ddrA* (*dr0423*), *ssB* (*dr0099*), *recQ* (*dr1289*), *ddrO* (*2574*) itself and so on (Fig. 3). ChIP-PCR assay with cell lysate confirmed that DdrO does bind to the promoter regions of these genes *in vivo* (Fig. 4). All these genes were found in previous studies to be up-regulated, after radiation in the presence of PprI [5–7], [11] and all their promoter regions contain the predicted radiation and desiccation resistance motif (RDRM) [13], which spreads in the upstream of many radiation-induced genes in *D*. *radiodurans* or in *D*. *geothermalis* [13]. Our subsequent result indicted that DdrO could bind nearly all promoter regions of DNA damage response genes with RDRM site, but the binding was destroyed by the presence of PprI (S5 Fig.). To verify the importance of RDRM site, DdrO binding to the promoter regions absent of the 17-bp palindromic RDRM sequence were tested, and it was found that shortened promoter regions fail to bind DdrO (Fig. 5). The minimum sequence for DdrO to bind in the promoter of *recA* is 5' TGTTATGCTGCTAGCAGAAAT 3', four deoxyribonucleic acids more than the RDRM sequence (underlined) (Fig. 6). Thus, RDRM is necessary for the specific binding of DdrO to its targets.

### An uncleavable mutant of *ddrO* sensitive to DNA damage

**Fig 7:**
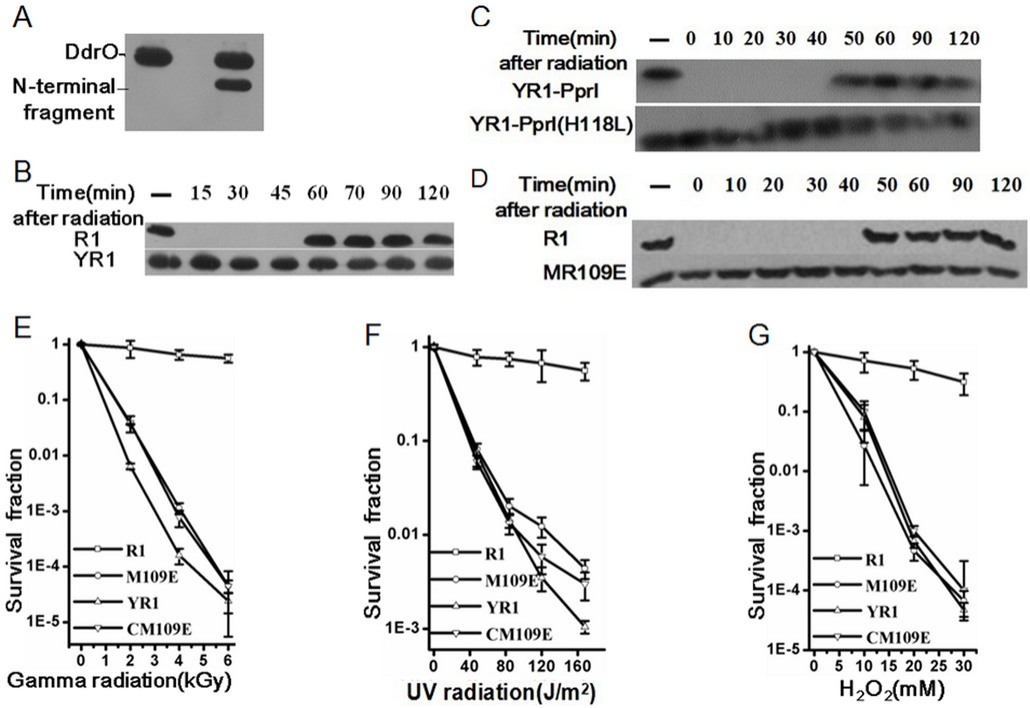
The uncleavable mutant strain MR109E. (A) The proteolytic product *in vitro* was assayed by western blotting. The total or N-terminal fragment of the cleaved substrate could be recognized by purified rabbit anti-DdrO polyclonal antibody. (B) A time-course-level of DdrO in the wild type R1 and YR1 mutant after gamma radiation assayed by western blotting. The first lane from left was the sample prior to ionizing radiation. The DdrO band of wild type R1 disappeared immediately after radiation and reappear one hour later, while the band of YR1 was constantly present. (C) A time-course-level of DdrO in the *pprI* complemented strain YR1-PprI and the *pprI* site-mutant YR1-PprI(H118L). (D) A time-course-level of DdrO in the wild type R1 and MR109E mutant after gamma radiation. The bands for MR109E remain unchanged after irradiation, similar to the result of YR1. (E) Survival rate of *D*. *radiodurans* under gamma radiation, (F) UV radiation and (G) H_2_O_2_ stress. R1 (open rectangle), wild type strain; MR109E (open circular), *ddrO* uncleavable mutant strain; YR1 (open triangle), *pprI*-knockout strain; CMR109E (inverted triangle), strain MR109E with the pRADK containing of the wild type *ddrO* gene. Data are the mean of triplicate experiments (error bars indicate standard deviation [SD]).

The *ddrO* knockout is lethal in both *D*. *radiodurans and D*. *deserti*, as efforts to construct such knockout strain has failed in several labs including ours [15]. In order to investigate the *in vivo* function of DdrO, we designed another approach. We constructed a *ddrO*-site disruptant and designated MR109E in which the entire coding region of the *ddrO* gene has been replaced by the whole coding mutation of R109E with a streptomycin resistance cassette. Western blotting was then utilized to assay whether the cleavage of DdrO happens *in vivo*. In the wild type R1, the band corresponding to DdrO disappeared immediately after radiation and recovers after one hour (Fig. 7A and 7B). In contrast, in the *pprI*-knockout mutant YR1, the uncleavable DdrO mutant MR109E and the *pprI* site-mutant YR1-PprI(H118L), the levels of DdrO remain unchanged before and after radiation (Fig. 7B-D), which suggests that DdrO-R109E mutation binding to promoter regions (S6 Fig.) was also working *in vivo*. To further investigate the biological role of *ddrO in vivo*, the survival phenotypes of strain R1, YR1, MR109E were examined through the exposure to DNA damaging agents. The strain MR109E was extremely sensitive to gamma radiation, UV radiation and H_2_O_2_ (Fig. 7E-G). Remarkably, the survival rate of MR109E was approaching that of YR1, demonstrating that the DdrO cleavage is vital for DNA damage response and cell survival. It also suggests that DdrO is likely to be the main if not sole effector of PprI to regulate DNA damage response. Then we complemented the wild type entire *ddrO* gene into the MR109E mutant through shuttle vector pRADK, and it can not restore the resistance of MR109E to gamma radiation, UV radiation and H_2_O_2_ (Fig. 7E-G). This is expected because even if the wild type DdrO can bind to the promoter regions of DDR genes, once it is cleaved after irradiation and dissociates, the DdrO-R109E mutant will replace it at the binding site.

### RNA transcription controlled by the cleavage

**Fig 8:**
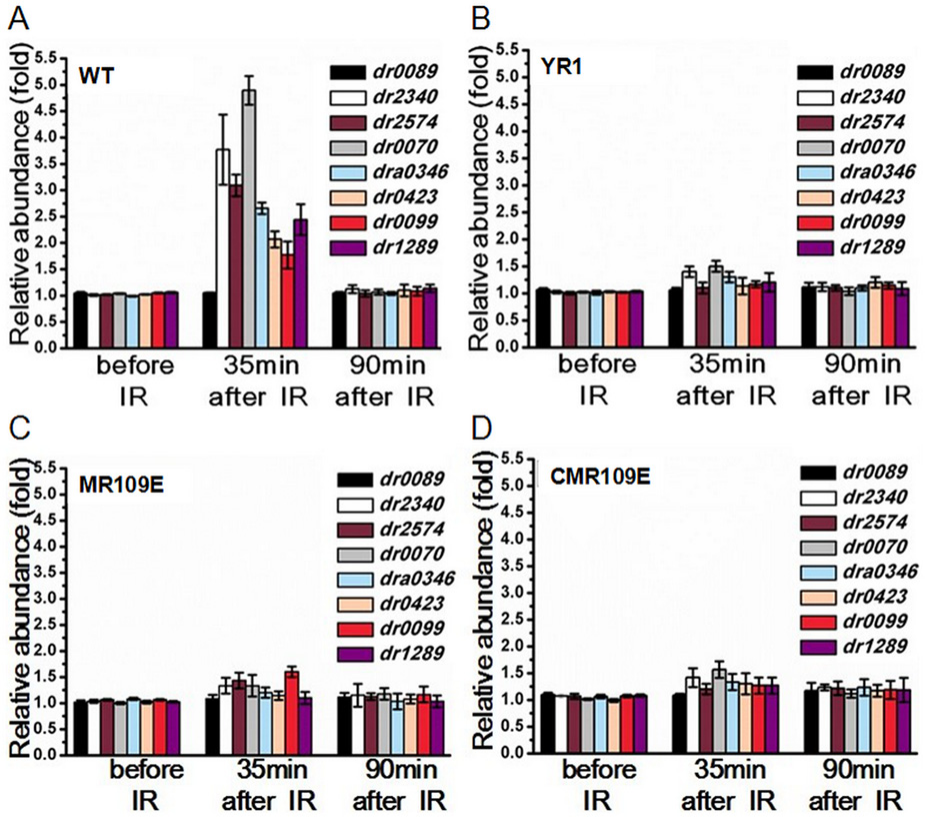
RNA transcription of *D. radiodurans* strains after exposure to gamma radiation. Total RNA was extracted and converted into cDNA using random 6 mers (Takara) and analyzed by QRT-PCR. All the data were based on three independent experiments. Three time points were chosen: before radiation, 35 minutes and 90 minutes after radiation. DDR genes were repressed after gamma radiation in the *pprI-*disruption strain YR1 and the uncleavable site-mutant strain MR109E on the transcriptional level. (A) Wild type R1; (B) YR1; (C) MR109E; (D) CMR109E.

We further treated strain R1, MR109E, YR1 and CMR109E with gamma radiation, collected samples at two different time points (35 min, 90 min) after incubation for recovery, and purified the RNA to analyze transcription of the chosen DDR genes. QRT-PCR analysis show that the transcription of DDR genes including *ddrO* itself in wild type strain R1 is upregulated significantly after exposing to radiation (Fig. 8A), while these genes have negligible change in the mutant strains YR1, MR109E and CMR109E (Fig. 8B-D). DDR genes were always repressed in the DdrO-uncleavable mutant MR109E and *pprI*-disrupted mutant YR1, indicating that mutant of *pprI* or *ddrO* had the same effect on the expression of the radiation-induced DDR genes, and the absence of DDR genes’ up-regulation is likely due to the failure of DdrO dissociation from the promoter of these genes in strain YR1 and MR109E. Therefore, under normal growth condition, RNA transcription of DDR genes are repressed by the stable binding of DdrO.

## Discussion

*Deinococcus radiodurans* is an important model organism valuable for the study of DNA repair. PprI protein has been implicated as a switch for DNA damage response. In this study, besides confirming the recent report that PprI can cleave a putative transcription factor DdrO [15], we explored the metal ion dependence and recognition sequence specificity of PprI. And we further investigated the function of *ddrO* in *D*. *radiodurans* by using a mutant strain where the native *ddrO* is replaced with an uncleavable version. Furthermore, the *in vivo* cleaved N-terminal of DdrO (N-DdrO) was not observed after radiation as well as the *in vitro* interaction between N-DdrO and PprI (S7 Fig. and S1 Protocol). These interesting results provide us with more insights into the mechanism by which *pprI* and *ddrO* orchestrate the DNA damage response.

Our results seem to show that the PprI cleavage of DdrO has a strict requirement for manganese ion as its metal cofactor. Not even weaker protease activity can be restored by other common divalent metal ions tested. In the PprI crystal structure, a zinc ion was observed at a conserved binding site only after soaking the crystal in ZnSO_4_ solution before freezing [12]. Therefore it is not clear from the structural work what the native metal ligand should be. Here we show that EDTA treatment abolishes the protease activity. Excess Mn(2+) was able to reconstitute PprI function while zinc ion can not. While a secondary metal binding site can not be ruled out, the fact that residues coordinate the zinc site is crucial for enzyme activity strongly suggests that Mn(2+) bind to this conserved site. *Deinococcus radiodurans* is known to accumulate very high intracellular manganese and low iron level comparing to radiosensitive bacteria [20–22]. The high manganese concentration was suggested to be essential for relieving oxidative stress and protecting proteins from damage caused by reactive oxygen species [22, 23].

Given the abundance of Mn(2+) in the *D*. *radiodurans* cells, it is not surprising that PprI has adopted Mn(2+) as its metal co-factor. The unanswered question here is whether Mn(2+) plays any role in the activation of PprI. One can speculate that PprI binds metal ion other than Mn(2+) before stress and thus protease activity was inhibited. When DNA damage occurs, the relative affinity of metal ions change and Mn(2+) displaces other ions at the binding site, thus activating PprI. The change in metal affinity can be caused by protein conformational change brought on by factors such as post translational modification or ligand binding in the GAF-like domain. This is a hypothesis that needs to be further investigated.

DdrO is annotated as XRE family transcription regulator protein with a predicted HTH (helix-turn-helix) domain for binding to the target. It was speculated to be a general regulator for DDR, because microarray assays show that it is the only up-regulated putative transcription factor at low gamma dose (3 kGy) [14]. The results we report here have put this hypothesis on solid experimental ground. After a comparative analysis of the *Deinococcus radiodurans* and the closely related *Deinococcus geothermalis*, Marakova et. al. identified a common palindromic DNA motif, designated as the Radiation /Desiccation Response Motif [13], in a conserved set of genes associated with resistance. Ludanyi *et al* confirmed that DdrO represses the RDRM-containing promoter of *ddrD* in *E*. *coli* through promoterless *lacZ* reporter gene [15]. In this study, we are able to show the direct binding of DdrO to the promotor region of RDRM containing DDR genes using gel mobility shift assay. Here we proved that 21 of the 24 DDR genes with RDRM sequence [13] are binding substrates of DdrO, while the other three ones with lower score do not bind. In addition, when RDRM sequence is deleted, the binding shift diminished. We further have obtained experimental evidence that DdrO could directly bind to the minimum RDRM sequence. So it is likely that DdrO regulates its targets through binding to or dissociating from RDRM sequence.

In bacteria such as *Escherichia coli*, the SOS response is activated after exposure to ultraviolet, ionizing radiation or chemical mutagens [24–26]. However, the classic SOS response system does not exist in *Deinococcus radiodurans* [27, 28]. Although two LexA homologs (Dra0344 and Dra0074) were found in this bacterium, neither of them regulates DNA damage response [27, 28]. In this study, the transcription factor DdrO, which shares no sequence homology with LexA, was found to play a role analogous to LexA in DDR. DdrO binds to the operator region of many essential genes involved in the DNA repair process, including itself. Different from LexA, DdrO does not cleave itself in the presence of RecA, single strand DNA, double strand DNA and ATP (S8 Fig.). Instead, PprI acts as the enzyme that cleaves DdrO following DNA damage. PprI can cut DdrO at a specific site near the carboxy terminal in the presence of manganese ions *in vitro*. The truncated DdrO then dissociates from its DNA target. This proteolytic reaction also happens *in vivo* after ionic radiation. In the mutant where PprI was absent, this cleavage does not take place, further confirms that PprI is the enzyme that cuts DdrO. Mutation that renders DdrO uncleavable to PprI sensitizes the bacterium towards ionic radiation, UV and peroxide. Microarray and proteomics data reported by several groups including us all show that the seven genes under DdrO control are up-regulated after irradiation [7, 11, 14]. Given that, DdrO binding seems to repress gene expression and its cleavage removes this repression. This is expected from the comparison to LexA. Both PprI and DdrO only exist in the *Deinococcus-Thermus* phylum. Based on these findings, we propose that the PprI and DdrO are the key players in this novel DNA damage response pathway.

**Fig 9:**
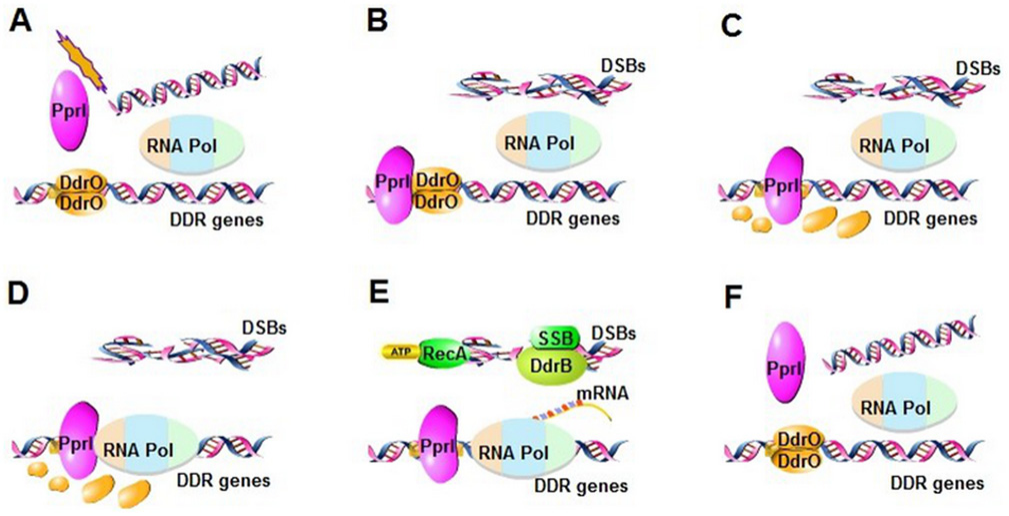
A hypothetical model of DNA damage response pathway mediated by PprI and DdrO. (A) Under normal growth condition, DDR genes are inhibited through the binding of DdrO to the promoter regions of DDR genes. (B-C) Once DNA is damaged, PprI is activated to bind to the promoter regions of DDR genes and cleave DdrO into two fragments. (D-E) Resulting in anti-oxidation, DNA repair, metabolic regulation and so on. (F) Some cells survive, in which DdrO rebinds to the promoter regions, and the transcriptions of DDR genes are stopped.

To summarize the proposed model (Fig. 9): Through some currently unknown mechanism, various DNA damage activates PprI (Fig. 9A), which in turn cleaves DdrO (Fig. 9B-C) and relieves the repression on many genes including those important for DNA repair. Those genes, including RecA, SSB and DdrB, carry out various processes of DNA damage repair (Fig. 9D-E). When DNA damage is fixed, PprI deactivates and DdrO re-establishes the suppression of the DDR genes (Fig. 9F).

While the pathway we described here shares some similarities to the classic SOS response, there are some important differences. The protease activity is shifted to a new player, PprI, from the transcription repressor itself. This affords more control points to the process. In the SOS pathway, the signal that triggers the response is the single strand DNA [29, 30]. It is unlikely that this is the case in DR, because PprI does not bind ssDNA and is active without ssDNA bound *in vitro*. The mechanism by which PprI is activated is still not clear. The reason that ssDNA-RecA filament is not used for signaling may be related to the observation that in *D*. *radiodurans* RecA binds preferentially to dsDNA over ssDNA therefore the ssDNA-RecA filament is not readily formed after DNA damage [31–33]. So the PprI-mediated DNA damage response pathway described here is likely to have evolved independent of the SOS pathway. Whether similar regulation mechanism of DDR exists in other organism or not is an intriguing question that warrants further investigation.

## Supporting Information

S1 Table Plasmids and strains. (DOC)

S2 Table Primers used in this study. (DOC)

S1 Protocol His-tag pull-down assay. (DOC)

S1 Fig Construction and confirmation of uncleavable mutant. (A) Schematic representation of the uncleavable mutant MR109E. AF1, AR1, AF2 and AR2 refers to primers, respectively. (B) The uncleavable mutant MR109E was confirmed by genomic PCR using AF1 and AR1. (C) The direct site-mutation of *ddrO* gene in mutant MR109E was checked by sequencing and sequence alignment.
(TIF)

S2 Fig Mass spectrometry analysis of the cleavage product. Molecular weight of the cleavage product was checked using SELDI-TOF-MS. The peak (15194.3+H) is the entire DdrO protein left after the cleavage, and the other peaks (12214.1+H, 3002.6+H) are the two cleaved fragments of DdrO, respectively.
(TIF)

S3 Fig Metal ion preference in PprI proteolytic reaction. (A) PprI protease activity does not change after incubating with metal ions for longer time. After incubation with 5 mM of metal ions for 2 hours, 8 μM of PprI was added and mixed with 450 μM of DdrO at 37°C for 1hour. (B) More DdrO was cleaved by increasing amount of PprI (0, 8, 16, 32 μM).
(TIF)

S4 Fig DdrO homologs from different organisms. Consensus amino acids and similar amino acids are shown in black and gray backgrounds, respectively. And the protease cleavage sequence is underlined.
(TIF)

S5 Fig PprI abolishes the binding of DdrO. GMSA was applied to detect PprI effect on the binding of DdrO to the promoter regions of *recA* and *ddrO*. The concentrations of promoters and DdrO are 1.6 μM, and 10 μM, respectively. With the increasing PprI concentration (0, 0.06, 0.3, 1.6, 8μM), the bands of DNA-protein complex were gradually weakened.
(TIF)

S6 Fig The uncleavable mutant of DdrO binds to DDR genes’ promoter regions. (A) The binding of the uncleavable mutant (DdrO-R109E) to the *recA* promoter region was analyzed by GMSA. The concentration of DNA is 1.6 μM and the DdrO concentrations are 0, 6, 8, 12μM, respectively. (B) GMSA for uncleavable mutant performed in different binding buffers(Mg(2+) or Mn(2+)) show no noticeable difference. The concentrations of DNA and DdrO are 0.8 and 5μM, respectively.
(TIF)

S7 Fig Identification of the interaction between PprI and N-terminal of DdrO (N-DdrO) through pull-down assay. 1, control blank of PprI; 2, control blank of N-DdrO; 3, the supernatant of the incubated mixture after short centrifugation; 4, the washing buffer of the agarose beads; 5, the eluted sample.
(TIF)

S8 Fig The relationship between RecA and DdrO. DdrO cleavage could not be activated by *Deinococcus radiodurans* RecA (Dr-RecA) and *Escherichia coli* RecA (Ec-RecA), even with the addition of ATP, single strand DNA (ssDNA), and double strand DNA (dsDNA). (A) Effect of EC-RecA on DdrO. (B) Effect of Dr-RecA on DdrO. CK1, a blank control of the boiled proteins before reaction; CK2, the reaction of RecA and DdrO; dsDNA, the reaction of RecA and DdrO with dsDNA and ATP; ssDNA, the reaction of RecA and DdrO with ssDNA and ATP. The reactions were incubated at 37°C for 1hour, then analyzed by SDS-PAGE.
(TIF)
