# Supplementary material for: Protease Activity of PprI Facilitates DNA Damage Response: Mn(2+)-Dependence and Substrate Sequence-Specificity of the Proteolytic Reaction
Source: PLoS One. 2015 Mar 26;10(3):e0122071. doi: 10.1371/journal.pone.0122071 (PMC4374696; doi:10.1371/journal.pone.0122071)
Supplement: S1 Table — (DOC) [file pone.0122071.s001.doc]

| **Plasmids and strains** | **Description** | **Source** |
| --- | --- | --- |
| **Plasmids** |  |  |
| pET28a(HMT) | pET28a derivative with a TEV protease site, a His tag and a maltose binding protein tag, Kan^R^ | Lab stock |
| pRADK | Shuttle plasmid between *E.coli* and *D. radiodurans*, Amp^R^, Kan^R^, Chl^R^ | Lab stock |
| pRADK-*ddrO* | pRADK containing wild type *ddrO* gene, Chl^R^, Amp^R^ | This study |
| pRADK-*pprI* | pRADK containing wild type *pprI* gene, Chl^R^, Amp^R^ | This study |
| pRADK-*pprI*(H118L) | pRADK containing H118L mutation of *pprI* gene, Chl^R^, Amp^R^ | This study |
| **Strains** |  |  |
| *E. Coli* |  |  |
| DH5α | Commercial | Trans Gen |
| BL21(DE3) | Commercial | Trans Gen |
| *D. radiodurans* |  |  |
| R1 | Wild type *D. radiodurans* strain (ATCC 13939) | Lab stock |
| YR1 | Disruptant of R1 deleted *pprI* gene, Kan^R^ | Lab stock |
| YR1-PprI | YR1 complemented with pRADK-*pprI*, Kan^R^, Chl^R^ | This study |
| YR1-PprI(H118L) | YR1 complemented with pRADK-*pprI*(H118L), Kan^R^, Chl^R^ | This study |
| MR109E | Disruptant of R1 with R109E mutation of *ddrO*, Str^R^ | This study |
| CMR109E | MR109E bearing pRADK-*ddrO*, Str^R^, Chl^R^ | This study |

**S1 Table. Plasmids and strains**
