# Supplementary material for: Protease Activity of PprI Facilitates DNA Damage Response: Mn(2+)-Dependence and Substrate Sequence-Specificity of the Proteolytic Reaction
Source: PLoS One. 2015 Mar 26;10(3):e0122071. doi: 10.1371/journal.pone.0122071 (PMC4374696; doi:10.1371/journal.pone.0122071)
Supplement: S2 Table — (DOC) [file pone.0122071.s002.doc]

**S2 Table. Primers used in this study**

**A. Primers for protein expression plasmids**

PprI F(NdeI): 5'GGAATTCATATGCCCAGTGCCAACGTCA3'

PprI R(BamHI): 5'CCGGGATCCGTCCAGTTCACTGTGC3'

DdrO F(NdeI): 5'GGAATTCATATGACATTGAAACTGCACGAACGACTTCGT3'

DdrO R(BamHI): 5'CCGGGATCCGGCCGGAGTTGTGCGGAAATCAGTT3'

**B. Primers for site-mutagenesis**

DdrO S104A F: 5'GGGTGCGGACCCTCGCGCGCATCGAGTTGC3'

DdrO S104A R: 5'GCAACTCGATGCGCGCGAGGGTCCGCACCC3'

DdrO R105A F: 5'TGCGGACCCTCTCGGCCATCGAGTTGCGTGGC3'

DdrO R105A R: 5'GCCACGCAACTCGATGGCCGAGAGGGTCCGCA3'

DdrO I106A F: 5'GGACCCTCTCGCGCGCCGAGTTGCGTGGCA3'

DdrO I106A R: 5'TGCCACGCAACTCGGCGCGCGAGAGGGTCC3'

DdrO E107A F: 5'CCTCTCGCGCATCGCGTTGCGTGGCAAGCG3'

DdrO E107A R: 5'CGCTTGCCACGCAACGCGATGCGCGAGAGG3'

DdrO L108A F: 5'TCTCGCGCATCGAGGCACGTGGCAAGCGC3'

DdrO L108A R: 5'GCGCTTGCCACGTGCCTCGATGCGCGAGA3'

DdrO R109A F: 5'GCGCATCGAGTTGGCTGGCAAGCGCCCGC3'

DdrO R109A R: 5'GCGGGCGCTTGCCAGCCAACTCGATGCGC3'

DdrO R109E F: 5'GCGCATCGAGTTGGAGGGCAAGCGCCCGC3'

DdrO R109E R: 5'GCGGGCGCTTGCCCTCCAACTCGATGCGC3'

DdrO G110A F: 5'TCGAGTTGCGTGCCAAGCGCCCGCGCG3'

DdrO G110A R: 5'CGCGCGGGCGCTTGGCACGCAACTCGA3'

DdrO K111A F: 5'AGTTGCGTGGCGCGCGCCCGCGCG3'

DdrO K111A R: 5'CGCGCGGGCGCGCGCCACGCAACT3'

DdrO K111E F: 5'AGTTGCGTGGCGAACGCCCGCGCG3'

DdrO K111E R: 5'CGCGCGGGCGTTCGCCACGCAACT3'

DdrO R112A F: 5'TGCGTGGCAAGGCCCCGCGCGACAAGCA3'

DdrO R112A R: 5'TGCTTGTCGCGCGGGGCCTTGCCACGCA3'

DdrO P113A F: 5'GCGTGGCAAGCGCGCGCGCGACAAGCA3'

DdrO P113A R: 5'TGCTTGTCGCGCGCGCGCTTGCCACGC3'

DdrO R114A F: 5'TGGCAAGCGCCCGGCCGACAAGCAGGACTGG3'

DdrO R114A R: 5'CCAGTCCTGCTTGTCGGCCGGGCGCTTGCCA3'

PprI H118L F: 5'TTCACCCTCGCCCTTGAAATCGGGCACGC3'

PprI H118L R: 5'GCGTGCCCGATTTCAAGGGCGAGGGTGAA3'

PprI E119Q F: 5'CCCTCGCCCACCAGATCGGGCACGCG3'

PprI E119Q R: 5'CGCGTGCCCGATCTGGTGGGCGAGGG3'

PprI H122L F: 5'CCCACGAAATCGGGCTCGCGATTTTACTCGGC3'

PprI H122L R: 5'GCCGAGTAAAATCGCGAGCCCGATTTCGTGGG3'

PprI E149Q F: 5'GCTCGAACAGGTCATCCAAACGCTGTGCAACGTG3'

PprI E149Q R: 5'CACGTTGCACAGCGTTTGGATGACCTGTTCGAGC3'

**C. Primers for construction of uncleavable mutant MR109E**

DdrO AF1: 5'CCCGGGCCGCCGACCCGGC3'

DdrO AR1(BamH ): 5'TTATAAGGATCCTCACCTCCTGGGCTGCGGCGGACC3'

DdrO AF2(Hind III) : 5'TTGTTGAAGCTTTTTCCGCACAACTCCGGCCCCGTC

G3'

DdrO AR2: 5'GCGCTGGCCTTTGCGGGTCTGCTCG3'

**D. Primers for qRT-PCR analysis**

*recA* RT F: 5'GCAAGGCCATCGAAACAGCC3'

*recA* RT R: 5'CAGTGCGAGGTCAAGGCTGA3'

*ddrO* RT F: 5'ACGACTTCGTGAATTGCGCAG3'

*ddrO* RT R: 5'GACCCCTTCGAGCAGGTCGT3'

*pprA* RT F: 5'TGGCAAGGGCTAAAGCAAAAGA3'

*pprA* RT R: 5'GCTTCTTGCAAGGACTGCGTGA3'

*ddrA* RT F: 5'TTCCCGCTCATACCGTGAGC3'

*ddrA* RT R: 5'TTCCGCACCGGACACCACTT3'

*ddrB* RT F: 5'TTATCACCGACCTGGGTGCG3'

*ddrB* RT R: 5'AAGTCGGCCTCGTTCTCGATG3'

*recQ* RT F: 5'TCAACTCGACGTTGCTGCCG3'

*recQ* RT R: 5'AAGTCGTGGCCCCACTGCG3'

*ssB* RT F: 5'ACCAAACGGACGGCATCTACG3'

*ssB* RT R: 5'TGTCGCCGTCGTCGGTCAG3'

*dr0089* RT F: 5'CGCTGCTGCTTTTTCCCATTC3'

*dr0089* RT R: 5'ACGGACCCGGTAGGCAAACT3'

**E. Primers for promoter regions**

P*dr1771* F: 5'GGGCGACCTGTTAGAATATTCCGT3'

P*dr1771* R: 5'TGGCTGTCCTGCACGCCTT3'

P*dra0151* F: 5'CTGTTCGACGGTGGCGAGG3'

P*dra0151* R: 5'ATCGTCCTCTGCGAGGCCC3'

P*dr2574* F: 5'GGCCAATTTCTGGTTTCGAAACA3'

P*dr2574* R: 5'CAGGCACCCTTGGTCGGAGT3'

P*dr0659* F: 5'GGGCGGGGAAGCGGG3'

P*dr0659* R: 5'TTGGCGGGTGCAAGGTTTG3'

P*dr1921* F: 5'TGGAAAACGAGCCCGCG3'

P*dr1921* R: 5'GCAACCGCCCGGCGT3'

P*dr0171* F: 5'CCCAGCAGGTTCTGCGGC 3'

P*dr0171* R: 5'GAGCCTGAGGGGGTGGACAC 3'

P*dr1262* F: 5'GGTTTGAGTCCCACCCGTCC3'

P*dr1262* R: 5'ACGTAAAGTCGGCAAACCGGT3'

P*dr1696* F: 5'CCCAACCTCCCGCCCG3'

P*dr1696* R: 5'CGTGGGGGGGGAGGACG 3'

P*dr1289* F: 5'AGGGGCGGGCCGGG3'

P*dr1289* R: 5'GGCAGGAGCAGCGGTCATCT3'

P*dr1775* F: 5'ACAAAAAGTCACGCCTAGCCCAA3'

P*dr1775* R: 5'GGAAGAAGTCACCGAAAGAGTTTAGCA3'

P*dr0070* F: 5'AGGTCTTGGTGCCCGGCC3'

P*dr0070* R: 5'TCAATCTGCAACATCTGCCTCCTC3'

P*dr0099* F: 5'GACCAAGAAGGCCTGAGCCTTTTA3'

P*dr0099* R: 5'TAGAGTTCGATGTTGCTGGCGG3'

P*dr0219* F: 5'CAGTCGCACGACTACTCATAACGAAA3'

P*dr0219* R: 5'TGTAGCACCCATGCTTAGGGATTATAA3'

P*dr1913* F: 5'TTTTCTTCGTCTTTTCCGGTAAACTG 3'

P*dr1913* R: 5'GGCACTCGCCGGGGGATA3'

P*dr0423* F: 5'CAGGGGCCGCCCCGA3'

P*dr0423* R: 5'CTCAGCTTCATGCATTTAGTTTAGAACAGA3'

P*dra0346* F: 5'GCCGAAGCTGGGTTTTCCTTATG3'

P*dra0346* R: 5'TGGTCTTTTGCTTTAGCCCTTGC3'

P*dr1143* F: 5'CGTGAGTCACAGCCCAGTGTGTC3'

P*dr1143* R: 5'TTCGTCATAGCTTCTCCTTTAAAACCC3'

P*dr0906* F: 5'CTAAAAGCCTCACTGCACCGAAGTT3'

P*dr0906* R : 5'AATATAGCGCTTGACACGAAAAATATCA3'

P*dr0326* F: 5'GCGCCCCTCTCCTGACTCG3'

P*dr0326* R: 5'GTTCCAGCTTTTTTCAGGGTATCCA3'

P*dr2256* F: 5'TACTCAAGTCACATTCACAGAGAGGCTT3'

P*dr2256* R: 5'CGGTCAGACTCGCAAGCGC3'

P*dr1039* F: 5'CCCCGTCTGGGGAGCGG3'

P*dr1039* R: 5'CTGCTCGCATAGAGGGACAAGATAGC3'

P*dr2275* F: 5'GCGCGCAGGCCGGG3'

P*dr2275* R: 5'TTCAAACAGAGTCTGAACGTCTGCC3'

P*dr0596* F: 5'CAGTACGGGGGCGGCTGAG3'

P*dr0596* R: 5'GGCGTCCAGATTCTCGGGG3'

P*dr2338* F: 5'CCGCACCCTGTAGACACTGGTTAC3'

P*dr2338* R: 5'CTCCAAACAGCAGTTCCGTGCC3'

P*recA* F: 5'AGAAACACCAGCATGATCGC3'

P*recA*- R: 5'CAACCTCACCGGAGTATATGG3'

P*recA* R: 5'CGTGCCCACACTGATGAT3'

P*ddrO* F: 5'TACTGCGGTCTGACGATGAC3'

P*ddrO*- R: 5'TGAGGGCAGTCAACTTGCCT3'

P*ddrO* R: 5'AGTGTAACACCGCTCCTTGC3'

P*ddrB* F: 5'GCACTGCGGCTTGCAGCGCC3'

P*ddrB*- R：5'CACATCGGGGAAGCCGGTGCC3'

P*ddrB* R: 5'AATCTGCAACATCTGCCTCCTCC3'

P*ddrA* F: 5'CGGGGCATAGGACGAAGGTAG3'

P*ddrA*- R: 5'AACGCCAGTTGATTAAGGGGAA3'

P*ddrA* R：5'AGTTTAGAACAGAATAACATTACGG3'

P*recQ* F：5'AGGAGCAGTTTCTTCATGAAGCC3'

P*recQ*- R：5'TCATGGAGGCCCCCCGCTG3'

P*recQ* R：5'CTCCCCAGGATAGCGCGTTTA3'

P*pprA* F：5'GTCACGGCCCCACTGTAGTCC3'

P*pprA*- R：5'TAAAAAGGCCAGGGGTAGCACT3'

P*pprA* R: 5'CCCTTGCCATACTGCCTTTATTAT3'

P*drssB* F: 5'CCAGGTGGGCAAGGACCGC3'

P*drssB*- R: 5'RAAGGCTCAGGCCTTCTTGGTC3'

P*drssB* R: 5'TAGTAACAAGCAGAGTCAATTATGT3'
