# Supplementary material for: Protease Activity of PprI Facilitates DNA Damage Response: Mn(2+)-Dependence and Substrate Sequence-Specificity of the Proteolytic Reaction
Source: PLoS One. 2015 Mar 26;10(3):e0122071. doi: 10.1371/journal.pone.0122071 (PMC4374696; doi:10.1371/journal.pone.0122071)
Supplement: S1 Protocol — (DOC) [file pone.0122071.s003.doc]

**His-tag pull-down assay**

Possible interaction between PprI protein and N-terminal part of DdrO（N-DdrO, a.a. 1-108）were tested using His-tag pull-down assay. 20 µg of purified N-DdrO protein with N-terminal His-tag was incubated with Ni-NTA agarose beads in 1 ml of pull-down buffer (167mM NaCl, 20 mM Tris-HCl pH 8.0, 5% glycerol) at 4℃for 30 minutes. The supernatant was removed by centrifugation. Then the beads were washed twice, and mixed with 20 µg of PprI protein in 1 ml of pull-down buffer. After 1 hour of incubation, the beads were collected and washed. Pull-down buffer with 500 mM imidazole was used to elute the proteins and the results was analyzed using SDS-PAGE.
