# Supplementary figures and images for: Protease Activity of PprI Facilitates DNA Damage Response: Mn(2+)-Dependence and Substrate Sequence-Specificity of the Proteolytic Reaction
Source: PLoS One. 2015 Mar 26;10(3):e0122071. doi: 10.1371/journal.pone.0122071 (PMC4374696; doi:10.1371/journal.pone.0122071)

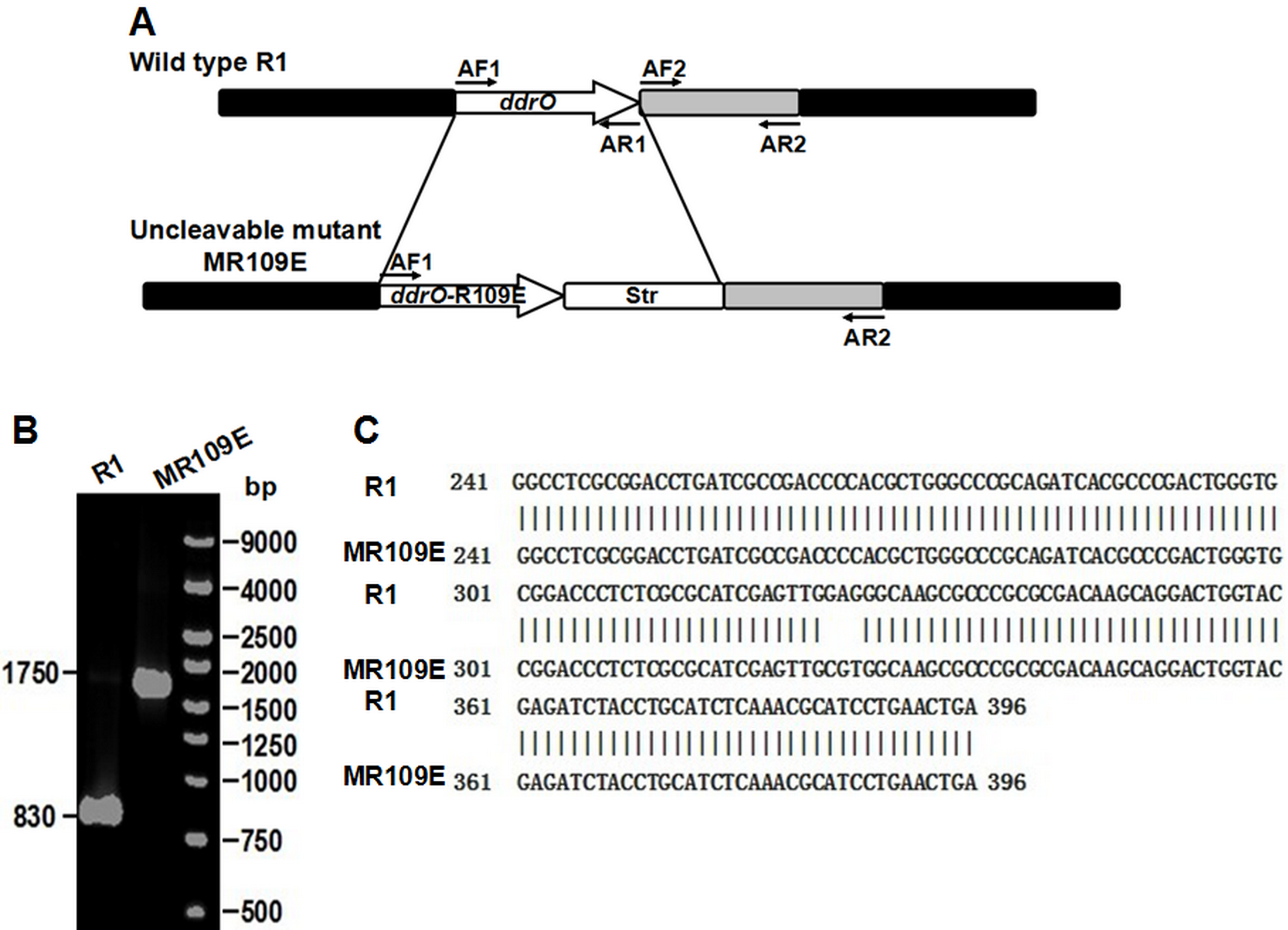

Supplement: S1 Fig — (A) Schematic representation of the uncleavable mutant MR109E. AF1, AR1, AF2 and AR2 refers to primers, respectively. (B) The uncleavable mutant MR109E was confirmed by genomic PCR using AF1 and AR1. (C) The direct site-mutation of ddrO gene in mutant MR109E was checked by sequencing and sequence alignment. (TIF) [file pone.0122071.s004.tif]

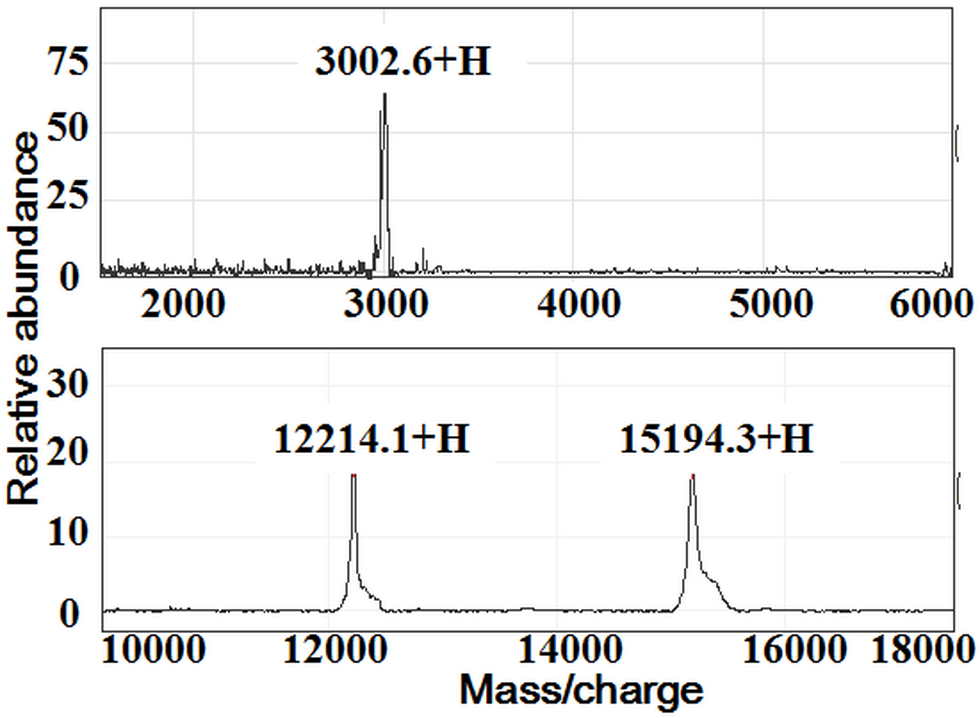

Supplement: S2 Fig — Molecular weight of the cleavage product was checked using SELDI-TOF-MS. The peak (15194.3+H) is the entire DdrO protein left after the cleavage, and the other peaks (12214.1+H, 3002.6+H) are the two cleaved fragments of DdrO, respectively. (TIF) [file pone.0122071.s005.tif]

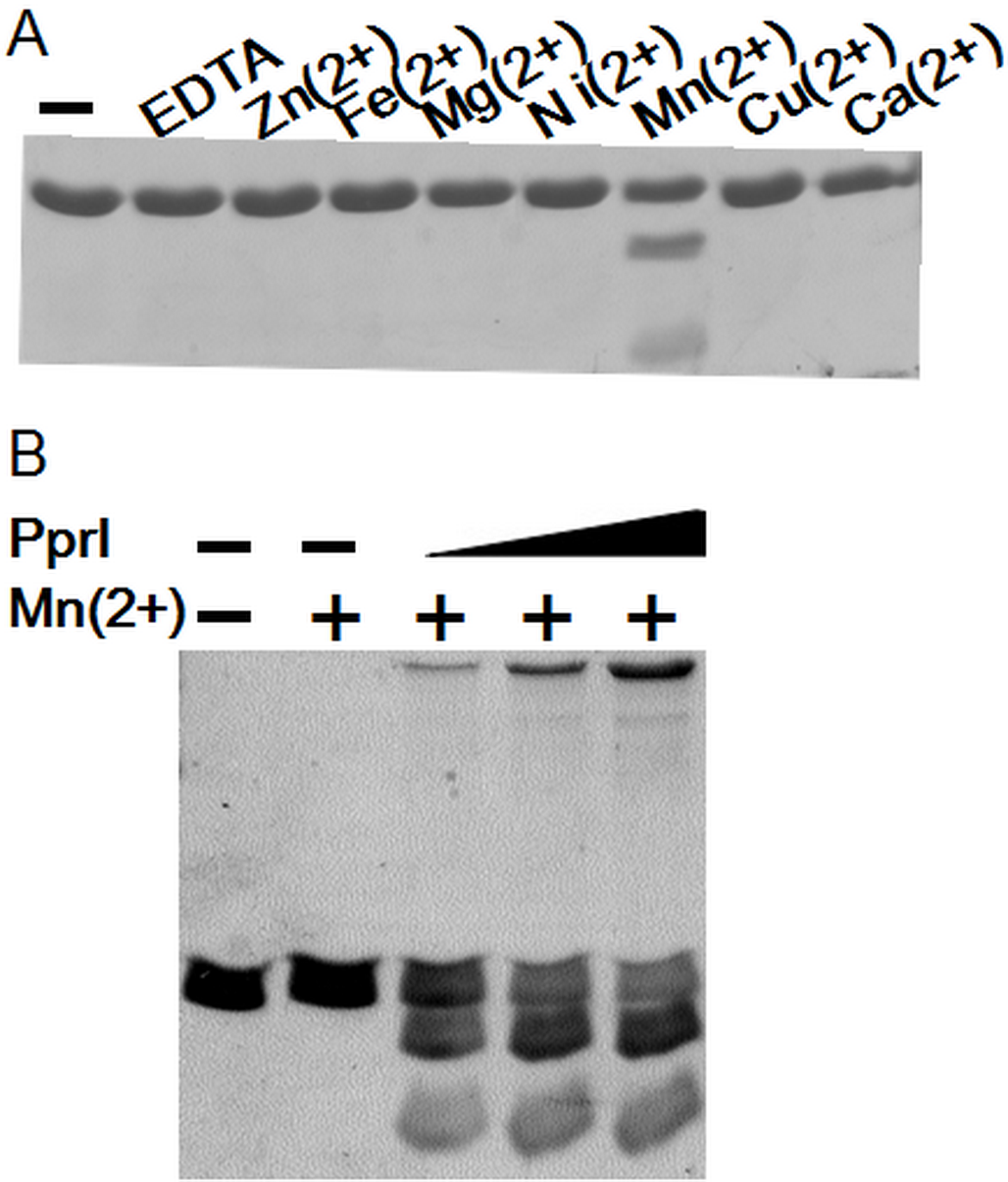

Supplement: S3 Fig — (A) PprI protease activity does not change after incubating with metal ions for longer time. After incubation with 5 mM of metal ions for 2 hours, 8 μM of PprI was added and mixed with 450 μM of DdrO at 37°C for 1hour. (B) More DdrO was cleaved by increasing amount of PprI (0, 8, 16, 32 μM). (TIF) [file pone.0122071.s006.tif]

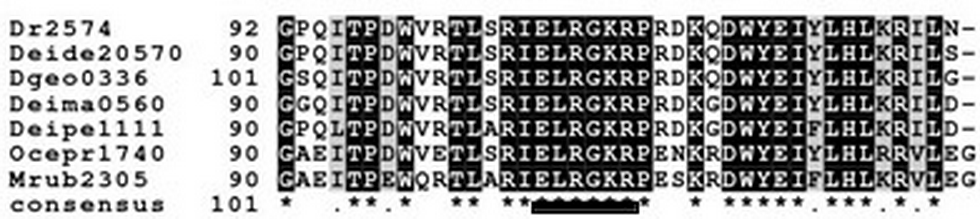

Supplement: S4 Fig — Consensus amino acids and similar amino acids are shown in black and gray backgrounds, respectively. And the protease cleavage sequence is underlined. (TIF) [file pone.0122071.s007.tif]

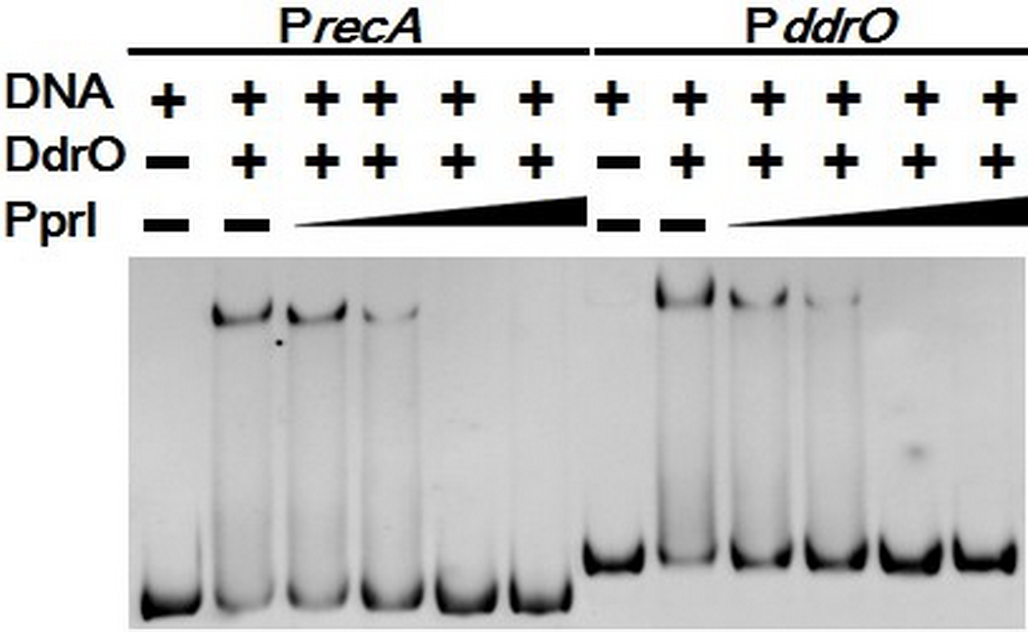

Supplement: S5 Fig — GMSA was applied to detect PprI effect on the binding of DdrO to the promoter regions of recA and ddrO. The concentrations of promoters and DdrO are 1.6 μM, and 10 μM, respectively. With the increasing PprI concentration (0, 0.06, 0.3, 1.6, 8μM), the bands of DNA-protein complex were gradually weakened. (TIF) [file pone.0122071.s008.tif]

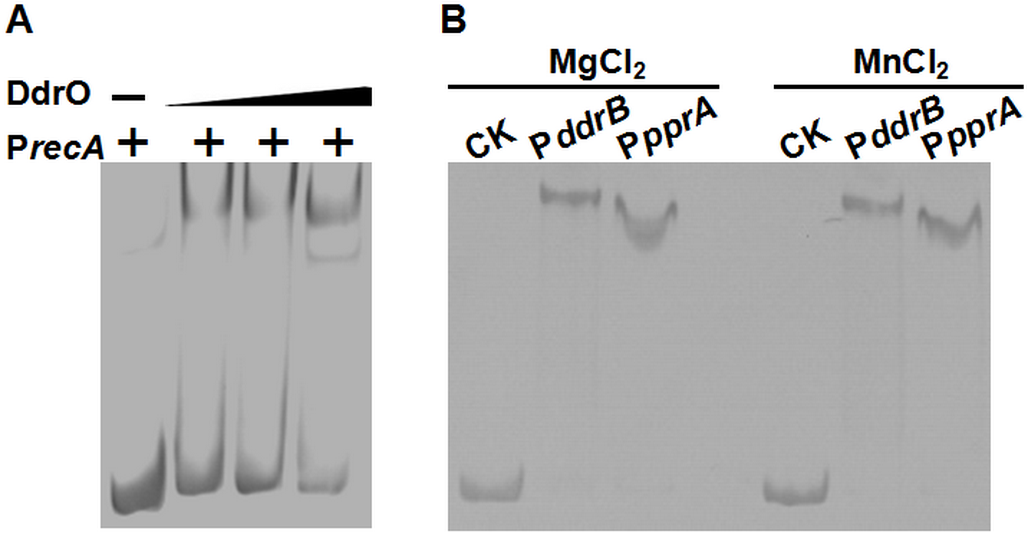

Supplement: S6 Fig — (A) The binding of the uncleavable mutant (DdrO-R109E) to the recA promoter region was analyzed by GMSA. The concentration of DNA is 1.6 μM and the DdrO concentrations are 0, 6, 8, 12μM, respectively. (B) GMSA for uncleavable mutant performed in different binding buffers(Mg(2+) or Mn(2+)) show no noticeable difference. The concentrations of DNA and DdrO are 0.8 and 5μM, respectively. (TIF) [file pone.0122071.s009.tif]

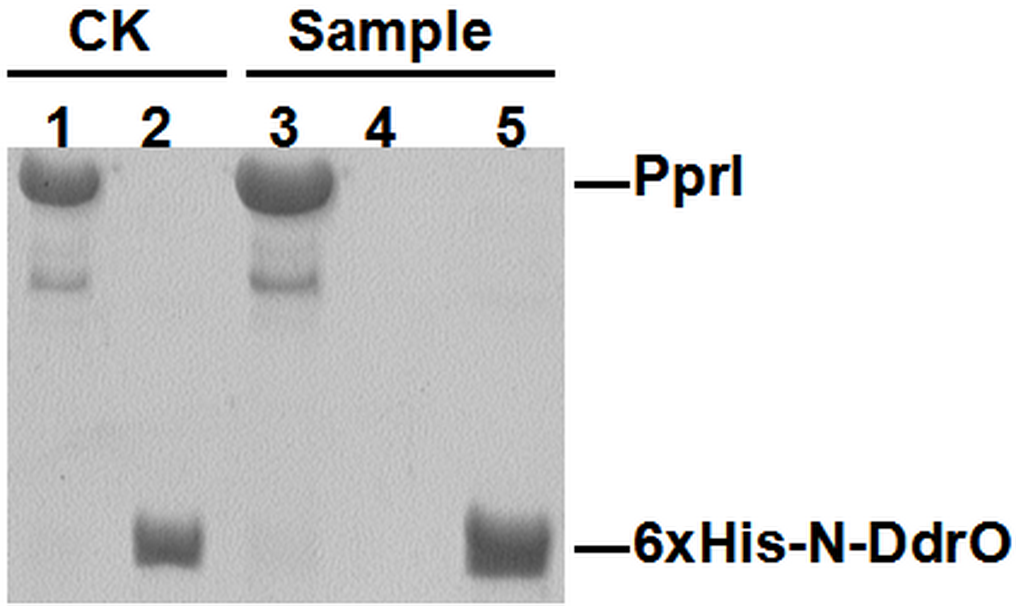

Supplement: S7 Fig — 1, control blank of PprI; 2, control blank of N-DdrO; 3, the supernatant of the incubated mixture after short centrifugation; 4, the washing buffer of the agarose beads; 5, the eluted sample. (TIF) [file pone.0122071.s010.tif]

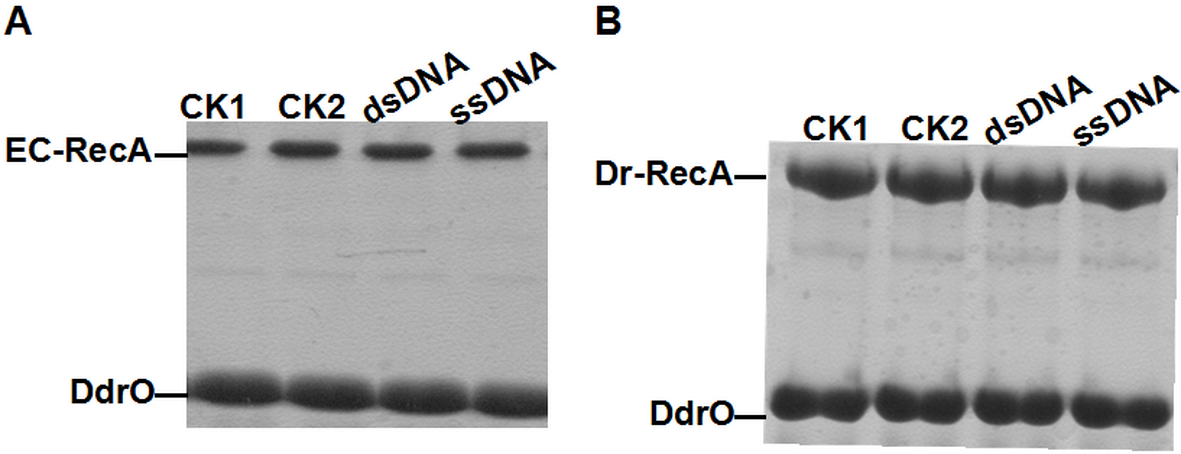

Supplement: S8 Fig — DdrO cleavage could not be activated by Deinococcus radiodurans RecA (Dr-RecA) and Escherichia coli RecA (Ec-RecA), even with the addition of ATP, single strand DNA (ssDNA), and double strand DNA (dsDNA). (A) Effect of EC-RecA on DdrO. (B) Effect of Dr-RecA on DdrO. CK1, a blank control of the boiled proteins before reaction; CK2, the reaction of RecA and DdrO; dsDNA, the reaction of RecA and DdrO with dsDNA and ATP; ssDNA, the reaction of RecA and DdrO with ssDNA and ATP. The reactions were incubated at 37°C for 1hour, then analyzed by SDS-PAGE. (TIF) [file pone.0122071.s011.tif]
